# Dynamic alterations in labile heme levels and heme biosynthesis during inflammatory activation of macrophages

**DOI:** 10.1016/j.redox.2026.104305

**Published:** 2026-07-16

**Authors:** Yan Jin, Pooja Pradhan, Hongxin Liang, Tomoyuki Nakagiri, Sebastian Mueller, Roberta Foresti, Roberto Motterlini, Tasuku Hirayama, Stephan Immenschuh

**Affiliations:** a Institute of Transfusion Medicine and Transplant Engineering, Hannover Medical School, Hannover, Germany; b Department of Cardiothoracic, Transplantation and Vascular Surgery, Hannover Medical School, Hannover, Germany; c Biomedical Research in Endstage and Obstructive Lung Disease Hannover (BREATH), German Center for Lung Research (DZL), Hannover, Germany; d Center for Alcohol Research, University of Heidelberg, Heidelberg, Germany; e University Paris-Est Créteil, INSERM, IMRB, Créteil, F-94010, France; f Laboratory of Chemical Biology, Gifu Pharmaceutical University, Gifu, 501-1196, Japan

## Abstract

Heme is an iron-containing tetrapyrrole with dual biological functions. While it serves as an essential prosthetic group in various hemoproteins, heme is cytotoxic in its ‘free’, non-protein-bound, form. Labile heme (LH) denotes the intracellular fraction of bioavailable heme that is readily exchangeable for incorporation into hemoproteins. To investigate the regulatory role of this heme fraction in inflammatory activated macrophages, we applied the selective fluorescent small molecule H-FluNox for LH detection in lipopolysaccharide (LPS)-stimulated murine bone marrow-derived macrophages (BMDMs). Studies with H-FluNox and its cell-permeable derivative acetylated (Ac)-H-FluNox revealed a time-dependent decrease of LH levels in living BMDMs upon treatment with LPS. Expression of δ-aminolevulinate synthase 1, the rate-limiting enzyme of heme synthesis, was up-regulated in parallel to decreased LH. Studies in subcellular organelles of BMDMs demonstrated that LH concentrations were markedly higher in mitochondria compared to cytosol and nuclei. Furthermore, expression of inducible nitric oxide synthase (iNOS), a heme-containing pro-inflammatory enzyme, was linked to intracellular LH concentrations. Specifically, LPS-dependent iNOS induction was attenuated in BMDMs displaying decreased LH, either after treatment with pharmacological heme synthesis inhibitors, or with genetic deficiency of the nuclear heme sensor BACH1. By contrast, inducibility of iNOS by LPS was markedly higher in BMDMs exhibiting increased levels of LH following treatment with the heme synthesis substrate δ-aminolevulinate. Finally, pharmacological inhibition of succinate dehydrogenase, which enhances intracellular levels of δ-aminolevulinate and LH, was also associated with higher inducibility of iNOS by LPS. In conclusion, the data indicate that intracellular LH is modulated by inflammatory stimulation in mouse macrophages and is critical for heme incorporation into the hemoprotein iNOS. Thus, heme availability may serve as a regulatory link between metabolic and inflammatory pathways.

## Highlights

• Intracellular levels of labile heme are decreased in inflammatory macrophages.
• Labile heme regulation and heme biosynthesis via ALAS1 are mutually interrelated.
• Expression of iNOS is dependent on labile heme levels in inflammatory macrophages.

## Graphical abstract

**Figure:**
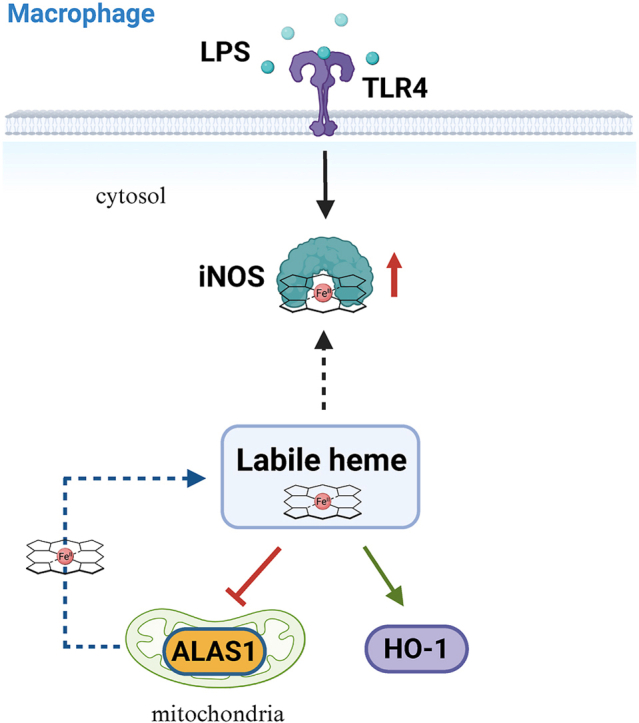

## 1 Introduction

Heme is a complex of protoporphyrin and iron with essential functions as a prosthetic group of oxygen- and electron-binding proteins, such as hemoglobin and cytochrome *c* [[1], [2], [3], [4], [5], [6]]. In contrast, ‘free’ (i.e. non-protein-bound) heme can exist inside and outside cells under particular conditions, exerting harmful effects via its pro-oxidant, pro-inflammatory and cytotoxic activities [[7], [8], [9]]. The toxicity of extracellular heme is counteracted by serum heme-binding proteins, such as hemopexin and albumin, which scavenge heme in pathophysiological conditions including hemolysis and ischemia-reperfusion injury [10,11]. By contrast, much less is known how intracellular heme toxicity and homeostasis are controlled in various cell types and conditions [[12], [13], [14]]. An intracellular fraction of bioavailable heme, referred to as the labile heme (LH) pool, has been proposed to serve as an exchangeable source of heme for rapid incorporation into intracellular hemoproteins [[14], [15], [16]]. While the concept of LH has been known for some time [17], in depth studies have been hampered by a lack of feasible methods to detect intracellular LH. Recently, the fluorescent small molecule probe H-FluNox has been introduced and applied for specific detection of LH intracellularly [18,19]. Notably, H-FluNox and its acetylated cell-permeable derivative Ac-H-FluNox have enabled studies on LH levels in living cells [18].

In macrophages, a heterogeneous population of mononuclear cells, heme has been shown to participate in immunomodulatory functions [20,21]. Macrophages play a central role in innate immune responses and dynamically adjust their immunological profile across various stages of inflammation that are commonly categorized into pro- and anti-inflammatory states [[22], [23], [24]]. Extracellular heme can induce inflammatory macrophage activation via Toll-like receptor (TLR)-4 signaling [[25], [26], [27]], but regulatory functions of intracellular heme have remained largely unknown. The goal of the current study was to better understand the role of intracellular heme homeostasis in macrophages. To this end, we used H-FluNox for detection of LH in mouse bone marrow-derived macrophages (BMDMs) stimulated with the prototypical inflammatory activator lipopolysaccharide (LPS).

We report here that intracellular LH levels are down-regulated over time in LPS-treated BMDMs coinciding with an up-regulation of δ-aminolevulinate synthase 1 (ALAS1), the rate-limiting enzyme of heme biosynthesis [28]. Moreover, the dynamic fluctuation of intracellular LH levels correlates with LPS-dependent up-regulation of heme oxygenase (HO)-1, the inducible isoform of heme catabolism [29], and the heme-containing enzyme inducible NO synthase (iNOS), a classical pro-inflammatory marker [30,31]. These results suggest key roles for heme synthesis, transient fluctuations in LH levels, and heme degradation in the control of macrophage activation by inflammatory stimuli.

## 2 Results

### 2.1 Intracellular LH levels decrease over time in LPS-stimulated BMDMs

**Fig. 1:**
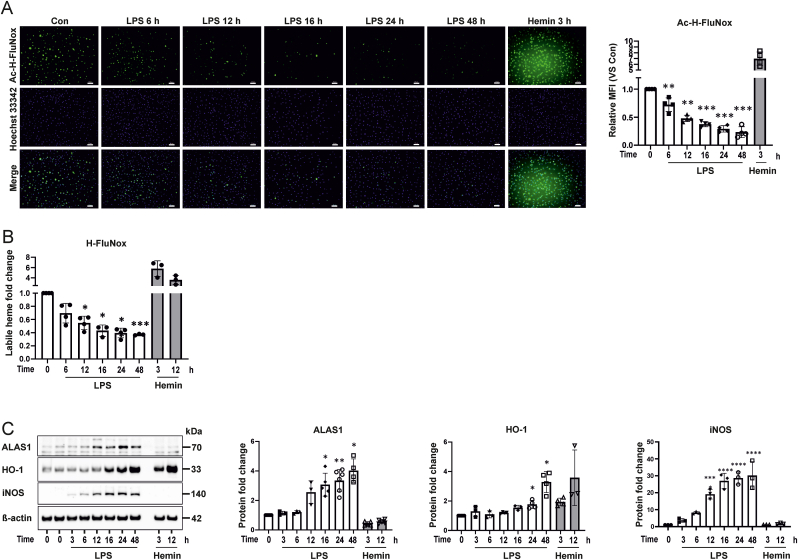
**LPS decreases LH levels and induces ALAS1 and HO-1 expression in BMDMs.** BMDMs were stimulated with LPS (1 μg/ml) for 6-48 h, with hemin (5 or 10 μM) as a positive control for LH. (A) Representative live-cell fluorescence images of Ac-H-FluNox (green) staining used to assess LH; nuclei were counterstained with Hoechst 33342 (blue). Scale bar, 50 μm. Right panel shows the corresponding quantification of mean fluorescence intensity (MFI). (B) H-FluNox fluorescence measured by a microplate reader and normalized to total protein content. (C) Western blot analysis and densitometric quantification of ALAS1, HO-1, iNOS, and β-actin in total cell lysates, normalized to β-actin. Data represent mean ± SD (n ≥ 3). Statistical significance was determined by one-way ANOVA followed by Tukey's post hoc test (*p < 0.05, **p < 0.01, ***p < 0.001, ****p < 0.0001). Ac-H-FluNox, acetylated H-FluNox; Con, control.

To better understand the immunomodulatory functions of heme in macrophages, we assessed the temporal changes of heme levels in these cells upon inflammatory activation with LPS using the fluorescent small molecule H-FluNox for detection of LH. LPS-treatment induced a time-dependent decrease of intracellular LH levels in living BMDMs over a period of up to 48 h, as determined with Ac-H-FluNox, the cell-permeable derivative of H-FluNox (Fig. 1A). By contrast, treatment with hemin increased LH levels (Fig. 1A). Similar results were obtained when LH was measured with H-FluNox in freshly prepared cellular extracts from BMDMs (Fig. 1B). In parallel, determination of total heme concentrations in LPS- and hemin-treated BMDMs revealed slightly increased heme levels following LPS stimulation of BMDMs and a marked increase in hemin-treated cells (Fig. S1). Thus, although LPS reduced the pool of LH, total cellular heme levels were modestly elevated under these conditions. We next examined the expression of proteins involved in heme metabolism. In LPS-stimulated BMDMs, the rate-limiting heme synthesis enzyme ALAS1 was up-regulated over time in parallel to the decline of LH levels. Moreover, expression of the heme-degrading enzyme HO-1 and that of the hemoprotein iNOS were also induced by LPS, although with distinct temporal kinetics (Fig. 1C). While expression of HO-1 was significantly increased only after 24 h, iNOS up-regulation was already detectable as early as 3 h after LPS stimulation (Fig. 1C). Taken together, these findings demonstrate that challenging BMDMs with LPS causes a time-dependent decrease in LH levels accompanied by a time-dependent up-regulation of ALAS1, HO-1 and iNOS expression.

### 2.2 ALAS1-dependent regulation of LH modulates the LPS response in BMDMs

**Fig. 2:**
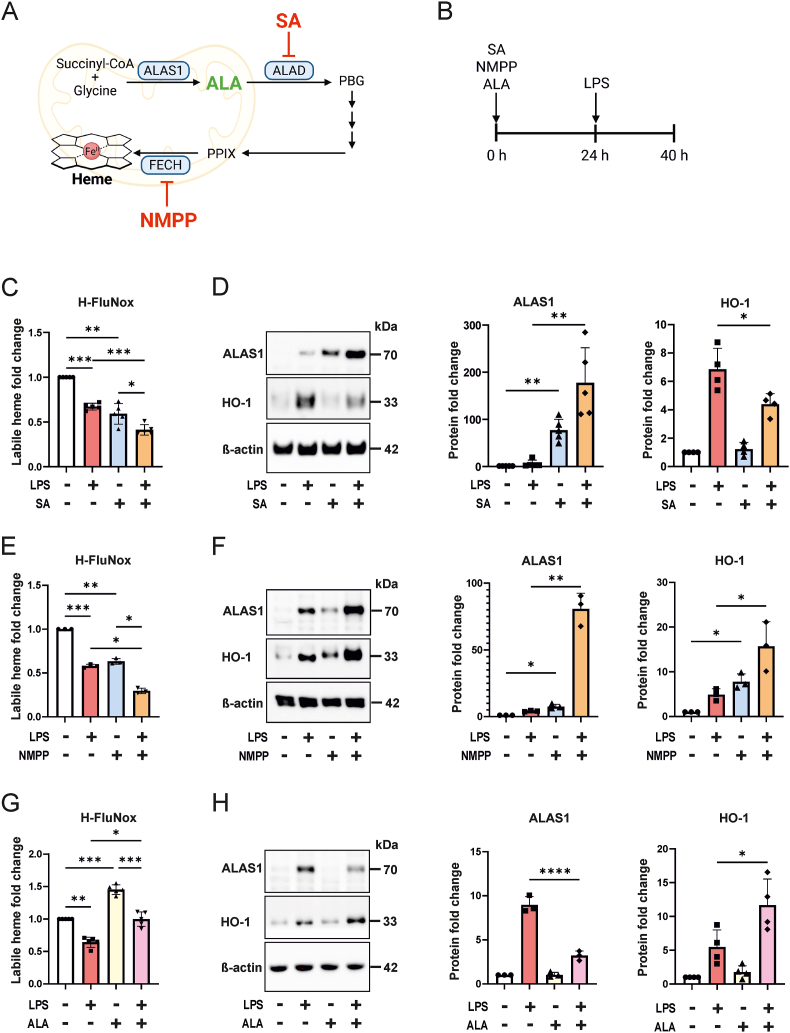
**Modulation of intracellular LH levels regulates ALAS1 and HO-1 expression in inflammatory macrophages.** (A) Schematic representation of the heme synthesis pathway highlighting the specific targets of inhibitors SA and NMPP, alongside the intermediate ALA (created with *BioRender.com*). (B) Experimental timeline and treatment strategy. BMDMs were pretreated with SA (2 mM), ALA (300 μM) or NMPP (5 μM) for 24 h, followed by LPS stimulation (1 μg/ml) for 16 h (C,E,G) Intracellular LH levels measured by H-FluNox fluorescence using a microplate reader and normalized to protein content relative to the control. (D,F,H) Western blot analysis and densitometric quantification of ALAS1, HO-1, and β-actin in total cell lysates, normalized to β-actin. Data represent mean ± SD (n ≥ 3). Statistical significance was determined by one-way ANOVA followed by Tukey's post hoc test (*p < 0.05, **p < 0.01, ***p < 0.001, ****p < 0.0001). ALA, δ-aminolevulinate; ALAD, ALA-dehydratase; FECH, ferrochelatase; NMPP, N-methyl protoporphyrin; PBG, porphobilinogen; PPIX, protoporphyrin IX; SA, succinylacetone.

As intracellular LH levels are dependent on ALAS1 activity [32], we wondered if modulation of ALAS1 enzyme activity could affect the LPS response in macrophages. To this end, ALAS1 was pharmacologically inhibited by succinylacetone (SA), an inhibitor of δ-aminolevulinate (ALA)-dehydratase, or N-methyl protoporphyrin IX (NMPP), an inhibitor of ferrochelatase (Fig. 2A). In parallel, experiments were conducted by activation of ALAS1 with its own substrate ALA (Fig. 2A). After pre-treatment of BMDMs with these compounds for 24 h, cells were cultured for an additional 16 h in the presence or absence of LPS (Fig. 2B). We observed that pretreatment with SA decreased LH levels and further enhanced LPS-dependent down-regulation of LH (Fig. 2C). As expected, in the absence of LPS, SA caused a marked up-regulation of ALAS1, while HO-1 remained unchanged (Fig. 2D). Notably, pretreatment with SA increased LPS-dependent ALAS1 expression in a synergistic manner and decreased the LPS-mediated HO-1 up-regulation. A similar effect on LH and ALAS1 protein expression was observed in BMDMs treated with NMPP (Fig. 2E and F). In contrast to SA, NMPP alone or in combination with LPS led to an up-regulation of HO-1 expression (Fig. 2D and F). When macrophages were pre-treated with ALA to provide the substrate for heme synthesis, a significant increase in LH was measured in unstimulated BMDMs. Interestingly, in LPS-challenged cells ALA prevented the reduction in LH levels, which remained similar to those of untreated cells (Fig. 2G). In concomitance, ALA reduced the up-regulation of ALAS1 mediated by LPS and enhanced LPS-dependent induction of HO-1 (Fig. 2H). Pretreatment with ALA and hemin counter-acted SA-induced expression of ALAS1 (Fig. S2). These data show that modulating the intracellular LH levels by either blocking (with SA or NMPP) or stimulating the enzymatic activity of ALAS1 (with ALA) affects the changes in LH levels elicited by LPS. Thus, it appears that the response of BMDMs to LPS is characterized by a reciprocal interaction between TLR4 signaling and endogenous LH via regulation of enzymatic heme synthesis and degradation.

### 2.3 LH and ALAS1 expression vary in subcellular compartments of LPS-stimulated BMDMs

**Fig. 3:**
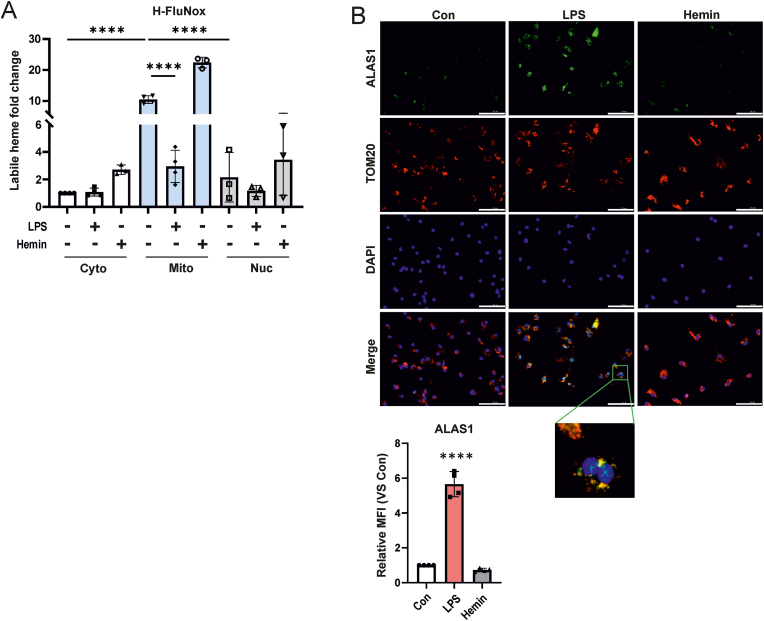
**LPS decreases mitochondrial LH and induces mitochondrial ALAS1 expression in BMDMs.** (A) H-FluNox fluorescence measured in cytoplasmic, nuclear, and mitochondrial fractions isolated from BMDMs following treatment with LPS (1 μg/ml, 16 h) or hemin (10 μM, 3 h). Values were normalized to cytosolic control based on protein content. (B) Representative immunofluorescence images of BMDMs treated with LPS (1 μg/ml, 16 h) or hemin (10 μM, 3 h), stained for ALAS1 (green), TOM20 (red), and nuclei (DAPI, blue) to assess mitochondrial localization. Representative images (scale bar, 50 μm) and corresponding mean fluorescence intensity (MFI) normalized to control based on cell number are shown. Data represent mean ± SD (n = 4). Statistical significance was determined by one-way ANOVA followed by Tukey's post hoc test (****p < 0.0001). Con, control; Cyto, cytosol; Mito, mitochondria; Nuc, nuclei.

Intracellular trafficking of heme is mediated by various transporter and carrier-dependent pathways [33] and the signaling action of LH may be dependent on its localization within macrophages. To determine whether LPS not only affects LH content, but also its endogenous distribution, we evaluated LH levels in distinct subcellular compartments, that is, mitochondria, nuclei and cytosol isolated from LPS-stimulated BMDMs. Purity of the subcellular fractions was confirmed using marker proteins for cytosol (β-actin), mitochondria (VDAC1) and nuclei (lamin B1), respectively (Fig. S3). Firstly, we observed that in resting BMDMs, LH levels were markedly higher in mitochondria compared to nuclei or cytosol. However, upon stimulation with LPS, LH levels were consistently diminished in mitochondria and nuclei, but remained unchanged in the cytosol (Fig. 3A). For a comparison, treatment with hemin caused a marked increase of LH in all subcellular compartments (Fig. 3A). We also determined ALAS1 expression and localization by immunofluorescence analysis and found that in LPS-treated BMDMs ALAS1 protein levels were markedly increased and co-localized with the mitochondrial marker protein TOM20 (Fig. 3B). In contrast, treatment of BMDMs with hemin did not change ALAS1 expression in mitochondria.

These findings demonstrate that in normal conditions BMDMs display an uneven cellular LH distribution, with higher levels in mitochondria relative to cytosol and nuclei. Furthermore, these data highlight that the reduction of LH measured in LPS-challenged macrophages is restricted to mitochondria and to a smaller extent to the nuclei.

### 2.4 LH and ALAS1 expression in BACH1-deficient BMDMs

**Fig. 4:**
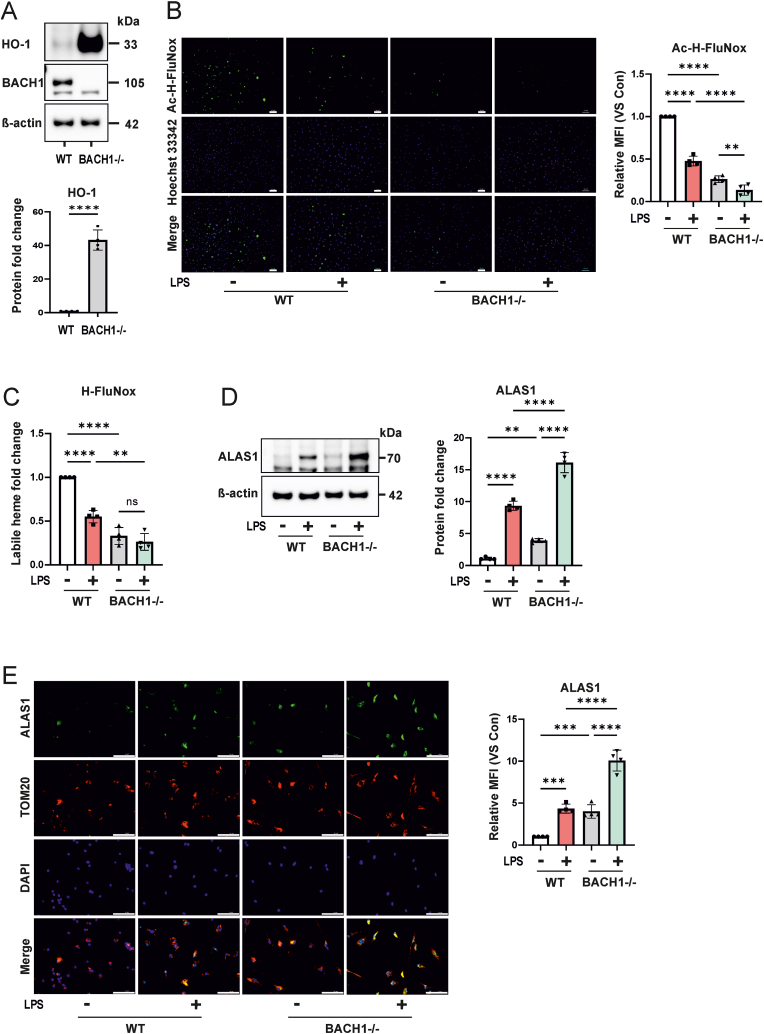
**BACH1 deficiency reduces LH levels and induces ALAS1 expression.** Wild-type and BACH1^−/−^ BMDMs were stimulated with LPS 1 μg/ml for 16 h. (A) Western blot analysis and densitometric quantification of HO-1 in total cell lysates, normalized to β-actin. (B) Representative live-cell fluorescence images stained with Ac-H-FluNox (green) to assess LH; nuclei were counterstained with Hoechst 33342 (blue). Scale bar, 50 μm. Right panel shows quantification of mean fluorescence intensity (MFI) normalized to wild-type control. (C) H-FluNox fluorescence measured by a microplate reader and normalized to wild-type control based on total protein content. (D) Western blot analysis and densitometric quantification of ALAS1 and β-actin in total cell lysates, normalized to β-actin. Note: The lower strong band on the ALAS1 blot is due to non-specific antibody binding. (E) Representative immunofluorescence images stained for ALAS1 (green), TOM20 (red), and nuclei (DAPI, blue). Scale bar, 50 μm. Corresponding ALAS1 fluorescence intensity quantification is shown. Data represent mean ± SD (n ≥ 3). Statistical significance was determined by one-way ANOVA followed by Tukey's post hoc test (**p < 0.01, ***p < 0.001, ***p < 0.0001). Con, control; WT, wild-type.

BTB and CNC homology 1 (BACH1) is a heme sensor protein and nuclear repressor of HO-1 gene expression [34]. In fact, exposure of cells to heme abrogates BACH1 repressor activity, leading to high induction of HO-1. Because both BACH-1 and enzymatic degradation of heme by HO-1 are tightly coordinated in the control of intracellular heme homeostasis, we determined LH levels and ALAS1 expression in BMDMs isolated from BACH1^−/−^ mice and compared the results with that of wild-type cells. Unsurprisingly, HO-1 expression was markedly higher in BACH1^−/−^ BMDMs than in wild-type cells (Fig. 4A). Studies with Ac-H-FluNox demonstrated that intracellular LH levels were lower in both untreated and LPS-treated BACH1^−/−^ cells compared to wild-type BMDMs (Fig. 4B). These findings were confirmed with H-FluNox in cell extracts of BACH1^−/−^ BMDMs (Fig. 4C). Also, in this setting we corroborated the strong correlation between LH and ALAS1 expression, showing that in untreated and LPS-treated BMDMs ALAS1 protein was higher in mitochondria (double-staining for ALAS1 and TOM20) of BACH1^−/−^ cells compared to wild-type (Fig. 4D and E). Taken together, BACH1^−/−^ BMDMs that constitutively express high HO-1 exhibit low LH levels in association with high expression of ALAS1.

### 2.5 LH affects LPS-dependent iNOS induction in BMDMs

**Fig. 5:**
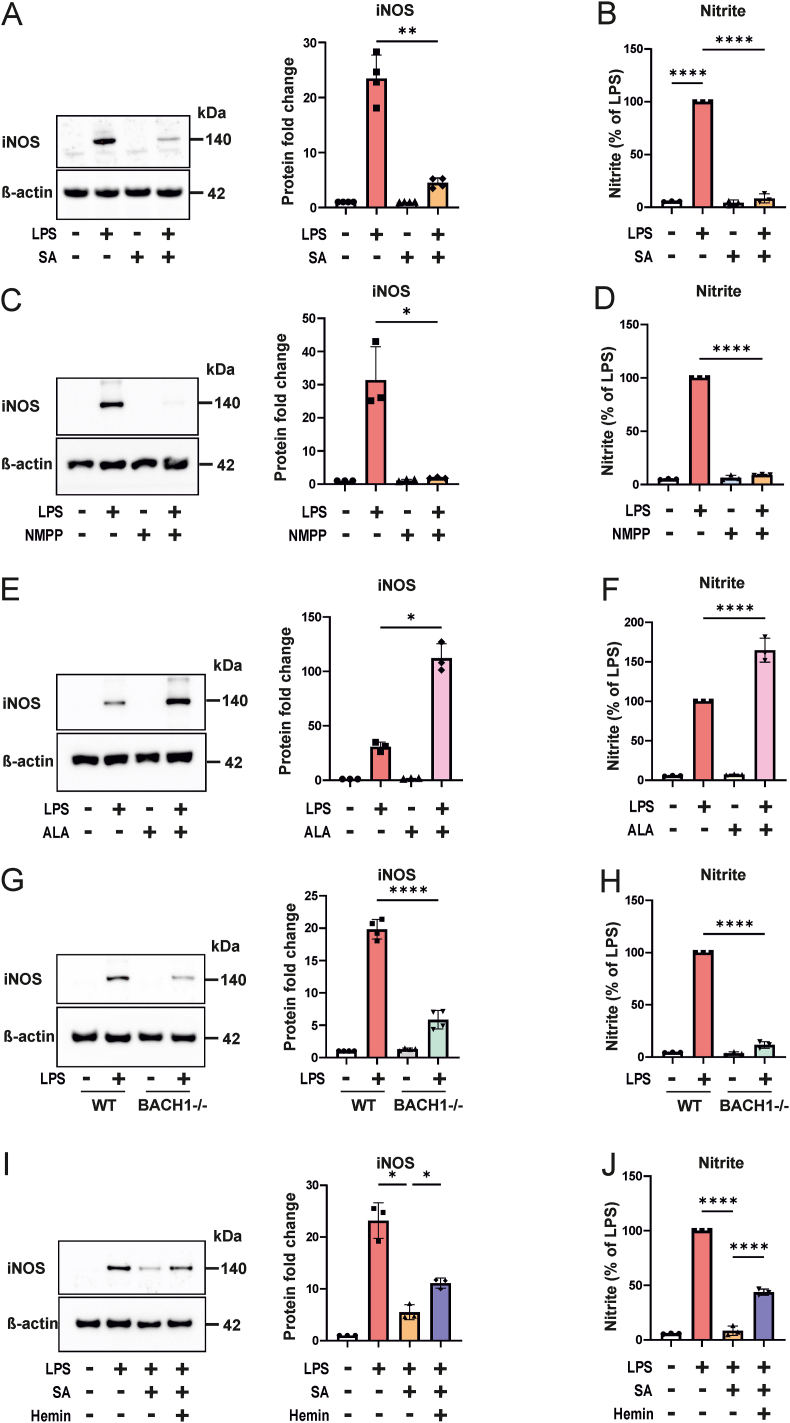
**Reduced LH levels attenuate LPS-induced iNOS expression and activity in BMDMs.** (A-F) Wild-type BMDMs were pretreated with SA (2 mM), ALA (300 μM) or NMPP (5 μM) for 24 h prior to LPS stimulation (1 μg/ml, 16 h). (A, C, E) Western blot analysis and densitometric quantification of iNOS and β-actin in total cell lysates, normalized to β-actin. (B, D, F) Nitrite production measured by microplate assay, with data normalized to protein concentration relative to LPS-treated controls. (G, H) Wild-type and BACH1^−/−^ BMDMs were stimulated with LPS alone (1 μg/ml, 16 h). (G) Western blot analysis and densitometric quantification of iNOS, normalized to β-actin. (H) Nitrite assay. (I, J) Wild-type BMDMs were pretreated with SA (2 mM) for 24 h before LPS (1 μg/ml) or hemin (1 μM) treatment (16 h). (I) Western blot analysis and densitometric quantification of iNOS, normalized to β-actin. (J) Nitrite assay. Data represent mean ± SD (n ≥ 3). Statistical significance was determined by one-way ANOVA followed by Tukey's post hoc test (*p < 0.05, **p < 0.01, ***p < 0.001, ****p < 0.0001). ALA, δ-aminolevulinate; NMPP, N-methyl protoporphyrin; SA, succinylacetone; WT, wild-type.

iNOS is a heme-containing protein that is strongly up-regulated in inflammatory activated mouse macrophages resulting in increased production of NO. Because *de novo* synthesis and functional activity of iNOS requires heme, we postulated that the intracellular LH pool might be used for this process. Thus, we next determined iNOS expression and nitrite levels in LPS-treated BMDMs containing low or high levels of LH. As in previous experiments, low levels of LH were achieved by pretreatment with SA and NMPP. In these conditions LPS-stimulated BMDMs exhibited a dramatic decrease of iNOS expression and nitrite production relative to BMDMs containing normal LH levels (Fig. 5A–D). By contrast, induction of interleukin (IL)-6 and tumor necrosis factor (TNF)-α, two prototypical pro-inflammatory cytokines, were up-regulated by lowering LH (Fig. S4A and S4B), indicating that LH modulates specific and selective LPS responses in macrophages. To further examine the impact of LH, BMDMs were pretreated with ALA to raise LH and we observed a significantly enhanced induction of iNOS and nitrite production by LPS (Fig. 5E and F). Once again, no effect of ALA pretreatment was found on IL-6 and TNF-α regulation (data not shown). Finally, in BACH1^−/−^ BMDMs, which exhibit reduced levels of LH (Fig. 4A and B), LPS-dependent iNOS induction and nitrite production were also attenuated (Fig. 5G and H). To further investigate the modulatory effects of exogenous heme, BMDMs were supplemented with hemin after pretreatment with SA to reconstitute the LH pool. Exposure to hemin partially restored iNOS induction by LPS in SA-pretreated BMDMs (Fig. 5I and J). Taken together, these findings indicate that alterations of intracellular LH along with ALAS1 activity are crucial for *de novo* synthesis of iNOS in LPS-stimulated macrophages, while other LPS-dependent responses (secretion of IL-6 and TNF-α release) exhibit opposite effects.

### 2.6 Inhibition of succinate dehydrogenase (SDH) causes an increase of LH in BMDMs

**Fig. 6:**
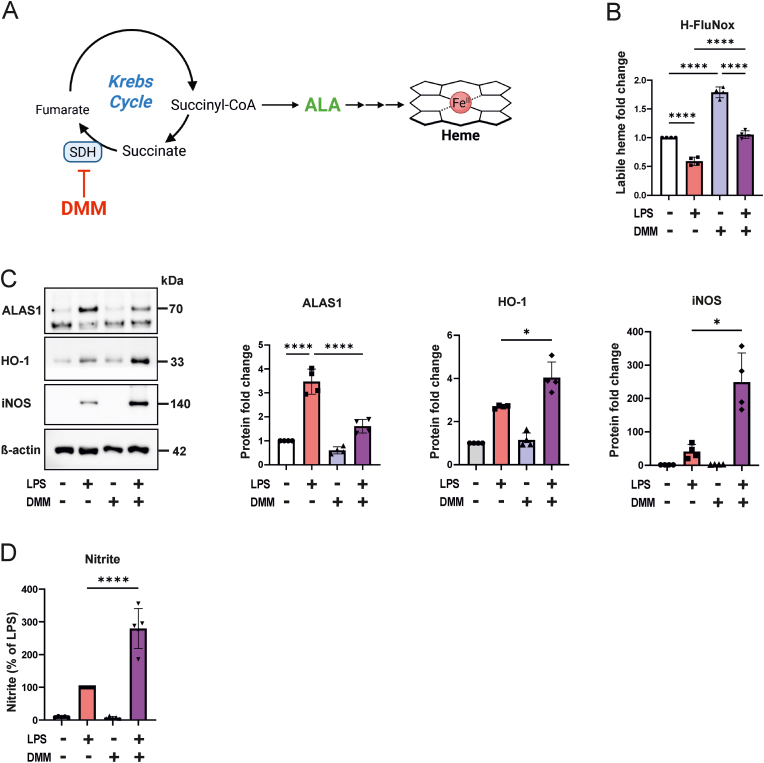
**SDH inhibition enhances LH levels and iNOS expression in BMDMs.** (A) Schematic representation highlighting the specific target of the inhibitor DMM (created with *BioRender.com*). (B) WT BMDMs were pretreated with DMM (5 mM) for 24 h followed by LPS stimulation (1 μg/ml, 16 h). Intracellular labile heme levels measured by H-FluNox fluorescence and normalized to total protein concentration. (C) Western blot analysis and densitometric quantification of ALAS1, HO-1, iNOS, and β-actin in total cell lysates, normalized to β-actin. Note: The lower strong band on the ALAS1 blot is due to non-specific antibody binding. (D) Nitrite production. Data represent mean ± SD (n ≥ 3). Statistical significance was determined by one-way ANOVA followed by Tukey's post hoc test (*p < 0.05, ***p < 0.0001). DMM, dimethyl malonate; SDH, succinate dehydrogenase.

LPS stimulation of mouse macrophages causes a shift of cellular bioenergetics in the glycolysis pathway and in mitochondria function [35]. This metabolic reprogramming is associated with inhibition of succinate dehydrogenase (SDH; mitochondrial complex II and the enzyme of the tricarboxylic acid (TCA) cycle that transforms succinate in fumarate), resulting in accumulation of succinyl-CoA (Fig. 6A). Because succinyl-CoA is the substrate of ALAS1, we investigated the effects of the pharmacological SDH inhibitor dimethyl maleate (DMM) as an approach to modulate LH in LPS-stimulated BMDMs. Exposure of unstimulated BMDMs to DMM caused a significant increase of LH without altering ALAS1 protein levels. Furthermore, the LPS-dependent decrease of LH was prevented in cells pretreated with DMM (Fig. 6B), concomitantly with a reduction in ALAS1 expression compared to LPS-treated cells. Moreover, HO-1 and iNOS expression as well as nitrite production were not affected by DMM in control BMDMs, but were significantly enhanced in LPS-challenged cells (Fig. 6C and D). By contrast, pretreatment with DMM did not enhance LPS-dependent up-regulation of IL-6 and TNF-α in BMDMs (Fig. S5A and S5B).

In summary, inhibition of SDH with DMM increases LH levels in BMDMs and enhances LPS-mediated iNOS induction. These findings show a strong interdependence between SDH activity and heme biosynthesis that may ultimately regulate the production of NO in inflammatory activated macrophages.

## 3 Discussion

Heme, an abundant iron-containing tetrapyrrole, is involved in immunomodulatory functions of macrophages and may bridge redox metabolism with immune activation. In the current study, we investigated how the dynamics of intracellular LH are modulated in inflammatory mouse BMDMs. Using the selective fluorescent probe H-FluNox, it is demonstrated that levels of LH are down-regulated in LPS-stimulated BMDMs. This effect is organelle-specific, because mitochondria exhibited higher LH levels than the nucleus and the cytosol in untreated cells, and the marked reduction of LH upon LPS stimulation was primarily observed in the mitochondria. Moreover, we show that the changes of LH elicit major modulation of ALAS1 and inflammatory iNOS expression in activated macrophages, revealing a close link between intracellular heme homeostasis and immune regulation.

The LH pool is the intracellular fraction of bioavailable heme that is loosely associated to proteins and lipids and can readily allocate into apo-hemoproteins [14,17]. A long-standing challenge in studies on LH regulation has been the lack of a feasible method for its detection [14,36]. In the present study, we applied the selective fluorescent compound H-FluNox to detect LH in resting and LPS-stimulated murine BMDMs, observing that LPS caused a time-dependent decrease of LH in living BMDMs (Ac-H-FluNox) and in corresponding cellular extracts (H-FluNox). The reduction in LH was accompanied by increased expression of the rate-limiting enzyme of heme biosynthesis, ALAS1 [17,32]. This result was expected and confirmed that H-FluNox is suitable for accurate detection of LH in primary cells. Unlike other heme detection methods that may disrupt cellular homeostasis, H-FluNox enables direct determination of heme bioavailability and visualization of LH changes during transition of macrophages from a resting to an inflammatory state. We previously applied an apo-HRP-based assay to measure LH in cellular extracts, but the cell lysis step required in this method likely caused denaturation of hemoproteins and a consequent artificial release of heme, potentially compromising the accuracy of our heme measurements [37,38]. Other methods of heme detection are available and have been applied for measurements of extracellular heme, e.g. in the serum of circulating blood [39]. However, most of these methods are not suited for determining intracellular LH. In addition, cellular LH can be determined via genetically encoded fluorescence sensor proteins that require cell transfection or transduction, but these approaches are not applicable for studies in primary mammalian cells such as BMDMs. A further limitation is that overexpression of fluorescent heme-binding proteins can skew intracellular LH levels by heme scavenging [33,[40], [41], [42]]. Thus, the use of the Ac-H-FluNox and H-FluNox probes provides a ‘clean’ approach for determining the fluctuations of LH during LPS challenge of macrophages. These probes also enabled us to clearly determine that LH levels are higher in mitochondria compared to the cytosol and nuclei. This finding is not unexpected as mitochondria are the site of heme synthesis and it can be predicted that a fraction of newly synthesized heme remains in a ‘free’ form until it is incorporated into hemoproteins. It was nevertheless interesting to observe that LPS markedly decreased mitochondrial LH, suggesting that inflammatory challenges induced a redistribution or degradation of LH within subcellular compartments. Notably, this loss of mitochondrial LH does not lower total cellular heme, as mitochondrial LH represents only a minor fraction of the total cellular heme pool. Our data suggest that the LPS-induced depletion is a highly localized event restricted to the mitochondria. The various experiments demonstrating that the LH pool could be dynamically changed by adding ALA or increasing its endogenous levels with the SDH inhibitor DMM, exogenous heme or SA, together with concomitant modulation of ALAS1 and HO-1, indicate that macrophages finely sense and respond to changes of free heme levels during inflammation. Although alterations in mitochondrial metabolism such as enhanced glycolysis and disrupted TCA flux have been established in LPS-stimulated macrophages [43,44], it is currently unknown, how fluctuations of LH may interact with these mitochondrial processes.

Macrophages are central players of the innate immune system with major functions for killing of invading pathogenic microorganisms and modulation of inflammatory responses [22,23]. Upon inflammatory stimulation, macrophages undergo major transcriptional and metabolic reprogramming that affects antimicrobial activity and cytokine production. Our current findings suggest that fluctuations in the cellular LH pool are an integral part of this response, which is in line with observations that stress-dependent signaling cascades are directly relevant for heme metabolism in inflammatory-activated macrophages. Indeed, NF-E2-related factor 2 (NRF2), the master regulator of the stress response that controls the expression of various inducible protective genes, including HO-1 and ferritin [45,46], plays a central role in these processes. Our data show that in macrophages challenged with LPS, ALAS1 is markedly increased from early (12 h) to late time points (48 h), whereas HO-1 up-regulation is manifested only at late times (24 and 48 h). This is consistent with the role of HO-1 in restoring cellular redox homeostasis. HO-1 actively contributes to these defense mechanisms thanks to: 1) enzymatic degradation of heme, which, at high levels, leads to increased generation of reactive oxygen species (ROS); and 2) the consequent production of biliverdin and carbon monoxide (CO), two protective metabolites possessing antioxidant and anti-inflammatory effects. In addition, heme-derived iron is sequestered by ferritin thereby limiting oxidative injury [47]. Regarding ALAS1, our results suggest a previously unrecognized role for this enzyme in the adaptation of macrophage to LPS challenge. This involves the replenishment of LH in the first phase after inflammatory stimulation and additional, yet unidentified mechanisms that may contribute to the resolution of inflammation at later time points. Overall, our findings indicate that a balanced control in heme synthesis, utilization, and degradation is critical for maintaining cellular redox homeostasis during inflammatory activation and macrophage polarization.

Low levels of LH are associated with decreased LPS-dependent inducibility of iNOS, an enzyme that produces large amounts of NO for antimicrobial defense [31,48]. Because heme is an essential cofactor for iNOS [30,49], it is plausible that heme from the LH pool is transferred and incorporated into *de novo* synthesized iNOS in inflammatory BMDMs. Our findings showing that decreased iNOS protein and NO generation in LPS-treated BMDMs exhibiting low LH after inhibition of heme biosynthesis suggest a strong mutual interrelation between heme availability and assembly of iNOS protein. The modulatory effects of LH were specific for iNOS, because the secretion of other inflammatory mediators such as IL-6 and TNF-α exhibited a distinct regulatory pattern. Similar findings have previously been reported for other hemoproteins such as NADPH oxidase (NOX)-5 and indoleamine 2, 3, dioxygenase (IDO) [16,36]. These observations reinforce the concept that LH is a heme source for functional hemoprotein maturation in inflammatory macrophages. We also explored the possibility that the nuclear repressor BACH1 is involved in these regulatory events, because BACH1 is an intracellular sensor of LH and binds antioxidant response elements (AREs) that are targeted by NRF2 to control the expression of HO-1 and other cytoprotective proteins. High levels of heme cause degradation of BACH1, thereby relieving repression and subsequent NRF2-dependent transcriptional activation. Changes in LH availability may therefore modulate the balance between BACH1-mediated repression and NRF2-driven stress responses that modulate inflammatory gene expression programs. Our results show that BACH1^−/−^ BMDMs contain significantly lower LH compared to wild-type cells, and LH is only marginally affected by LPS in these cells. This is most likely due to enhanced expression of HO-1 in BACH1^−/−^ macrophages, which may continuously consume LH as a substrate for its enzymatic activity. Interestingly, ALAS1 was higher in both untreated and LPS-challenged BACH1^−/−^ macrophages, indicating that an interdependence between this repressor, the LH pool and heme synthesis is required at the onset of inflammation. Thus, when macrophages sense that LH is below a certain threshold, they will compensate by increasing heme synthesis via ALAS1 induction. Importantly, the current findings confirm our previously published data that BACH1^−/−^ macrophages express lower iNOS protein compared to wild-type cells upon LPS stimulation [50]. The data also provide independent, non-pharmacological evidence that variations in the intracellular LH pool are directly linked to regulation and functional maturation of iNOS in inflammatory macrophages. It is important to distinguish the different actions of intra- and extracellular free heme. In fact, it is known that extracellular free heme acquires the characteristics of a damage-associated molecular pattern (DAMP) that induces pro-inflammatory activation not only in macrophages, but also in endothelial cells via TLR4 signaling [51]. By contrast, our present results show that intracellular LH decreases upon LPS stimulation, suggesting differential sensing and signaling activities of macrophages to endogenous and exogenous free heme.

In conclusion, the fluorescent small molecule H-FluNox affords detection of intracellular LH in macrophages. Its use enabled us to demonstrate that LPS stimulation down-regulates the LH pool, especially in mitochondria. Our findings support the existence of a tightly coordinated response in inflammatory macrophages that involves heme degradation and heme biosynthesis in a time-dependent manner. In particular, an increased expression of ALAS1, the rate-limiting enzyme of heme synthesis, likely reflects a necessary mechanism required to replenish endogenous heme during inflammation. We further identified the assembly of iNOS protein as a downstream target of the LH-ALAS1 axis coupled with BACH1 as a transcriptional regulator that contributes to the control of both LH and ALAS1 expression. Together, these findings provide a framework for understanding, how heme availability may modulate macrophage function and establish the basis for future studies exploring the networks connecting heme metabolism and immune responses.

## 4 Materials and methods

### 4.1 Reagents

All reagents, antibodies, instruments, and equipments, along with their respective catalog numbers and manufacturers, are listed in Supplementary Materials unless otherwise specified.

### 4.2 Mice

BACH1−/− mice were obtained from Tohoku University (Sendai, Japan) and backcrossed for more than 10 generations onto a C57BL/6J background at the Central Animal Facility of Hannover Medical School. Male mice aged 10-14 weeks were used for all experiments. Animals were maintained under specific pathogen-free conditions on a standard diet. All animal procedures were approved by the institutional Committee for Animal Welfare.

### 4.3 Isolation and cell culture of BMDMs

Bone marrow cells were isolated from tibiae and femora using a 22G needle and filtered through a 70-μm cell strainer. Cells were cultured for 7 days in high-glucose Dulbecco's Modified Eagle Medium (DMEM) supplemented with 10% fetal bovine serum (FBS), 1% penicillin-streptomycin, and 25 ng/ml recombinant mouse macrophage colony stimulating factor (M-CSF) to generate BMDMs. All treatments were performed in culture medium containing 1% FBS and 12.5 ng/ml M-CSF to minimize interference from serum-derived labile heme, as previously reported [33].

### 4.4 Protein isolation and quantification

Cells were washed with PBS and lysed using RIPA Lysis Buffer System using 10 μL of buffer per 1 × 10^5^ cells. The lysates were incubated on ice for 30 min and then centrifuged at 12,000 × g for 20 min at 4°C to pellet cellular debris. The supernatant was collected as the total protein lysate. Total protein concentration was quantified via a bicinchoninic acid (BCA) assay following the manufacturer's instructions. Briefly, BCA reagents A and B were mixed at a 50:1 ratio and added to the diluted samples and standards. Following a 30 min incubation at 60°C, absorbance was measured at 562 nm. Final protein concentrations were determined using the standard curve and subsequently used to normalize protein loading for Western blotting, nitrite assays, and H-FluNox measurements.

### 4.5 Measurement of intracellular labile heme with H-FluNox

LH was measured as described previously [18,19]. Following treatment, cells were washed with PBS and lysed using RIPA Lysis Buffer System. The lysates were incubated on ice for 30 min and centrifuged to collect the supernatant. To quantify LH, supernatant aliquots were incubated with 200 nM H-FluNox probe in PBS at 37°C for 30 min. Fluorescence intensity was then measured at excitation/emission wavelengths of 480/530 nm. All values were normalized to protein concentrations determined via the BCA assay.

### 4.6 Live-cell *in situ* imaging of intracellular LH levels using Ac-H-FluNox

Live-cell imaging of intracellular LH in BMDMs was performed as previously described [18]. Briefly, cells were washed three times with Hanks' Balanced Salt Solution (HBSS) and incubated in HBSS containing 30 μM Ac-H-FluNox and Hoechst 33342 at 37°C for 30 min. After four subsequent washes with HBSS, live-cell imaging was performed immediately using the FITC and DAPI channels of a BZ-X810 fluorescence microscope.

### 4.7 Total heme quantification

Intracellular total heme levels were determined using a commercial assay Kit (MAK316, Sigma-Aldrich) [52]. Following two PBS washes, cells cultured in 12-well plates were lysed directly in 200 μL of the provided heme reagent. The cell lysates and a series of heme standards, prepared in the same reagent, were transferred to a 96-well plate. Absorbance of was measured at 400 nm using a microplate reader. Final heme concentrations were calculated using a standard curve.

### 4.8 Nitrite assay

Nitrite levels, as an index of NO production, were measured using the Griess Reagent System according to the manufacturer's instructions. Briefly, sulfanilamide solution equilibrated to room temperature, was mixed 1:1 with either nitrite standards or undiluted cell culture supernatants and incubated for 5 min. An equal volume of NED Solution was then added, followed by incubation for 10 min. Absorbance was measured immediately at 535 nm. Nitrite concentrations were calculated from the resulting standard curve and normalized to the protein content determined by the BCA assay.

### 4.9 Western blot analysis

Western blotting was performed as previously described [50]. Total protein samples in RIPA buffer were prepared with LDS sample buffer and a reducing agent, then separated via SDS-PAGE using a Bio-Rad Tetra Vertical Electrophoresis Cell. Proteins were transferred to PVDF membranes using the Turbo Transfer System and its corresponding RTA kit. Membranes were blocked with EveryBlot Blocking Buffer and incubated overnight at 4°C with primary antibodies diluted in LowCross Buffer. The following day, membranes were washed four times for 5 min each with PBST (0.05% Tween-20 in PBS), and incubated with secondary antibodies for 1 h at room temperature. After four additional 5 min washes with PBST, proteins bands were visualized using either Clarity Max Western ECL Substrate or West Atto Ultimate Sensitivity Substrate and captured via a ChemiDoc MP Imaging System.

### 4.10 Immunofluorescence

For immunofluorescence analysis, cells cultured on coverslips were treated as indicated, fixed with 4% paraformaldehyde (PFA) for 20 min, and permeabilized with 0.3% Triton X-100 for 10 min. Cells were then blocked in PBS containing 1% bovine serum albumin (BSA) for 30 min and incubated overnight at 4°C with primary antibodies and the corresponding isotype controls. The next day, after washing, cells were incubated with secondary antibodies for 1 h at room temperature, washed again, and stained with DAPI for 5 min [38]. After three final washes with PBS containing 1% BSA, the coverslips were mounted onto glass slides using Fluoromount-W, sealed with nail polish, and imaged using a BZ-X810 fluorescence microscope.

### 4.11 Isolation by subcellular fractionation

Subcellular fractionation of BMDMs was performed using a rapid differential centrifugation protocol [53,54] adapted for low-biomass primary cells, yielding nuclear, mitochondrial, and cytosolic fractions within 90 min. Cells were homogenized on ice in a hypotonic buffer (210 mM mannitol, 70 mM sucrose, 5 mM MOPS, and 1 mM EDTA, pH 7.4) supplemented with protease inhibitors, using repeated passage through a 27.5-gauge needle. To isolate the nuclei, the crude homogenate was centrifuged at 800 × g for 15 min. The resulting nuclear pellet was washed in 0.1% NP-40/PBS and sedimented at 15,000 × g for 20 min to yield the final nuclear fraction.

The initial 800 × g supernatant was then centrifuged at 17,000 × g for 40 min to separate the mitochondrial and cytosolic fractions. The resulting supernatant was clarified by a brief centrifugation at 800 × g and collected as the cytosolic fraction. Meanwhile, the mitochondrial pellet was washed in 0.1% NP-40/PBS and pelleted at 15,000 × g for 15 min to obtain the mitochondrial fraction. Protein concentrations were quantified using a BCA assay. Fraction purity was validated by Western blot using antibodies against lamin B (nucleus), VDAC1 (mitochondria), and β-actin (cytosol).

### 4.12 Data processing and statistical analysis

Western blot images were quantified using ImageLab. Cell images were acquired with a BZ-X800 Viewer and analyzed or merged using ImageJ. Absorbance measurements were recorded and analyzed with Gen5, whereas H-FluNox fluorescence values were collected and analyzed using Magellan. Each experiment was performed independently at least three times. Statistical analyses were conducted using Student's t-test or one-way ANOVA followed by Tukey's post hoc test in GraphPad Prism 9, as previously described [27]. Data are presented as mean ± standard deviation.

## Funding

RF and RM were supported by the Fondation pour la Recherche Médicale (grant number EQU202103012568 to RF and RM), INSERM and University of Paris-Est, Créteil. TH was supported by 10.13039/501100001691Grant-in-Aid for Scientific Research (B) from the Japan Society for the Promotion of Science (JSPS KAKENHI, Grant number 23K23490 to TH). SI was supported by the 10.13039/501100000780European Union and the state of Niedersachsen (ZW 7-87011730).

## CRediT authorship contribution statement

**Yan Jin:** Data curation, Formal analysis, Investigation, Methodology, Validation, Writing – original draft. **Pooja Pradhan:** Conceptualization, Data curation, Formal analysis, Investigation, Methodology, Supervision, Validation, Writing – review & editing. **Hongxin Liang:** Investigation, Methodology. **Tomoyuki Nakagiri:** Methodology, Resources, Writing – review & editing. **Sebastian Mueller:** Conceptualization, Validation, Writing – review & editing. **Roberta Foresti:** Conceptualization, Methodology, Validation, Writing – original draft. **Roberto Motterlini:** Conceptualization, Methodology, Validation, Writing – original draft. **Tasuku Hirayama:** Conceptualization, Methodology, Writing – review & editing. **Stephan Immenschuh:** Conceptualization, Funding acquisition, Resources, Supervision, Writing – original draft.

## Declaration of competing interest

The authors declare that they have no known competing financial interests or personal relationships that could have appeared to influence the work reported in this paper.

## Data Availability

Data will be made available on request.
