# Supplementary material for: Dynamic alterations in labile heme levels and heme biosynthesis during inflammatory activation of macrophages
Source: Redox Biol. 2026 Jul 16;96:104305. doi: 10.1016/j.redox.2026.104305 (PMC13393713; doi:10.1016/j.redox.2026.104305)
Supplement: Multimedia component 1 [file mmc1.pdf]

SUPPLEMENTARY MATERIALS

Equipments

Trans-Blot® Turbo™ Transfer System	Bio-rad	Feldkirchen, Germany
Mini-PROTEAN® Tetra Vertical Electrophoresis Cell	Bio-rad	Feldkirchen, Germany
PowerPac™ Basic Power Supply	Bio-rad	Feldkirchen, Germany
Synergy™ 2 Multi-Mode Microplate Reader	Biotek	Winooski, Vermont, USA
Thermomixer Compact	Eppendorf	Hamburg, Germany
Refrigerated benchtop centrifuge 6-16KS	Sigma	Burlington, Massachusetts, USA
Mastercycler® nexus X2	Eppendorf	Hamburg, Germany
NanoDrop 1000 Spectrophotometer ND-1000	Thermo	Waltham, Massachusetts, USA
Fresco™ 17 Microcentrifuge	Thermo Fisher Scientific	Waltham, Massachusetts, USA
Megafuge ST Plus	Thermo Scientific	Waltham, Massachusetts, USA
StepOnePlus™ Real-Time PCR System	Applied Biosystems™	Waltham, Massachusetts, USA
Lynx 6000 Superspeed Centrifuge	Thermo Scientific	Waltham, Massachusetts, USA
Fluorescence Microscope BZ-X810	KEYENCE	Osaka, Japan
FACSCanto™ Flow Cytometry System	BD Biosciences	San Jose, CA, USA
NanoQuant Infinite M200	TECAN	Männedorf, canton of Zürich, Switzerland
ChemiDoc MP Imaging System	Bio-rad	Feldkirchen, Germany
Electrophoresis Power Supply E865	Consort	Turnhout, Belgium
horizontal kuroGEL Mini Plus 10	VWR	Radnor, Pennsylvania, USA
Arium 611UF Water Purifier	Sartorius-Stedim	Göttingen, Germany
CP225D-0CE analytical balance	Sartorius	Göttingen, Germany

Antibodies

		Catalog number	
ALAS-H	Santa Cruz Biotechnology	sc-137093	Dallas, Texas, USA
iNOS	Novus Biologicals	NB300-605	Centennial, Colorado, USA
BACH1	Proteintech	14018-1-AP	Rosemont, Illinois, USA
HO-1	Enzo Life Sciences	ADI-SPA-895	Ann Arbor, MI, USA
VDAC1	Proteintech	55259-1-AP	Rosemont, Illinois, USA
TOM20	Proteintech	11802-1-AP	Rosemont, Illinois, USA
Lamin B1	Santa Cruz Biotechnology	sc-6216	Dallas, Texas, USA
Beta Actin	Abcam	ab8227	Waltham, MA, USA
Purified Mouse IgG1, κ Isotype Ctrl Antibody	BioLegend	401401	San Diego, California, USA
Rabbit IgG isotype control	Abclonal	AC042	Wuhan, Hubei, China
Donkey anti-Rabbit Secondary Antibody, Alexa Fluor™ 568	Invitrogen	A10042	Carlsbad, CA, USA
Donkey anti-Rabbit Secondary Antibody, Alexa Fluor™ 488	Invitrogen	A-21206	Carlsbad, CA, USA
Donkey anti-Mouse Secondary Antibody, Alexa Fluor™ 594	Invitrogen	A-21203	Carlsbad, CA, USA
Donkey anti-Mouse Secondary Antibody, Alexa Fluor™ 488	Invitrogen	A-21202	Carlsbad, CA, USA
Rabbit Anti-Goat Immunoglobulins/HRP	Dako	P0449	Jena, Germany
Rabbit Anti-Rat Immunoglobulins/HRP	Dako	P0450	Jena, Germany
Goat Anti-Rabbit Immunoglobulins/HRP	Dako	P0448	Jena, Germany
Rabbit Anti-Mouse Immunoglobulins/HRP	Dako	P0260	Jena, Germany

Reagents and Chemicals

		Catalog number	
DMEM - high glucose	Sigma-Aldrich	D5796	Burlington, Massachusetts, USA
Penicillin-Streptomycin	c.c.pro	Z-13-M	Oberdorla, Germany
Trypsin-EDTA 10x	c.c.pro	Z-26-M	Oberdorla, Germany
FBS Good	PAN BIOTECH	P40-37500	Aidenbach, Germany
Dulbecco′s Phosphate Buffered Saline	Sigma-Aldrich	D8537	Burlington, Massachusetts, USA

Hanks' Balanced Salt Solution	Gibco	14025-050	Waltham, Massachusetts, USA
Accutase Cell Detachment Solution	Capricorn Scientific	ACC-1B	Ebsdorfergrund, Germany
Sterile water	PAN BIOTECH	P04-991500	Aidenbach, Germany
Trypan Blue solution	Sigma-Aldrich	T8154	Burlington, Massachusetts, USA
Dimethyl Sulfoxide for cell culture	AppliChem ITW Reagents	A3672,0100	Darmstadt, Germany
Hemin	Frontier Specialty Chemicals	H651-9	Logan, UT, USA
N-Methyl Protoporphyrin IX	Frontier Specialty Chemicals	NMP576	Logan, UT, USA
Dimethyl malonate	Sigma-Aldrich	136441	Burlington, Massachusetts, USA
5-Aminolevulinic acid hydrochloride	Sigma-Aldrich	A3785	Burlington, Massachusetts, USA
Succinylacetone	Sigma-Aldrich	D1415	Burlington, Massachusetts, USA
Recombinant Mouse Macrophage Colony Stimulating Factor (rm M-CSF)	ImmunoTools	12343117	Friesoythe, Germany
LPS-EB	Invivogen	tlrl-eblps	San Diego, CA, USA
NuPAGE™ MOPS SDS Running Buffer	Invitrogen	NP0001	Carlsbad, CA, USA
NuPAGE™ Sample Reducing Agent	Invitrogen	NP0009	Carlsbad, CA, USA
NuPAGE™ LDS Sample Buffer	Invitrogen	NP0007	Carlsbad, CA, USA
NuPAGE™ Antioxidant	Invitrogen	NP0005	Carlsbad, CA, USA
30% Acrylamide/Bis Solution, 29:1	Bio-rad	1610156	Feldkirchen, Germany
EveryBlot Blocking Buffer	Bio-rad	12010020	Feldkirchen, Germany
Stripping Buffer	CANDOR Bioscience	150 500	Wangen im Allgäu, Germany
ROTI®Free Stripping Buffer 2.2 plus	Carl ROTH	3337.1	Karlsruhe, Germany
LowCross-Buffer®	CANDOR Bioscience	100 500	Wangen im Allgäu, Germany
TEMED	Carl ROTH	2367.3	Karlsruhe, Germany
Spectra™ Multicolor Broad Range Protein Ladder	Thermo Scientific	26634	Waltham, Massachusetts, USA
TMB ONE	Kementec	4380A	Taastrup, Denmark
Triton™ X-100	Sigma-Aldrich	X100	Burlington, Massachusetts, USA

Tween® 20, Molecular Biology Grade	Promega	H5151	Madison, Wisconsin, USA
2-PROPANOL, LC-MS	J.T. Baker	9827.1	Center Valley, Pennsylvania, USA
Ethanol absolut	J.T. Baker	8025.1	Center Valley, Pennsylvania, USA
Fluoromount W	SERVA	21634,01	Heidelberg, Germany
Paraformaldehyd (PFA) 4 %, in PBS pH 7,2	MORPHISTO	10303,005	Offenbach am Main, Germany
Probumin® Bovine Serum Albumin Diagnostic Grade	Millipore	82-045-1	Burlington, Massachusetts, USA
DPBS Powder	Sartorius	BE15-512D	Göttingen, Germany
TRIS	Carl ROTH	4855.2	Karlsruhe, Germany
Glycine	Carl ROTH	3790.2	Karlsruhe, Germany
TRIS Hydrochlorid	Carl ROTH	9090.2	Karlsruhe, Germany
SDS ultra pure	Carl ROTH	2326.1	Karlsruhe, Germany
Ammoniumperoxodisulfat	Carl ROTH	9592.2	Karlsruhe, Germany
L-Glutathione reduced	Sigma-Aldrich	G4251	Burlington, Massachusetts, USA
3-Morpholinopropane sulfonic acid (MOPS)	Sigma-Aldrich	M1254	Burlington, Massachusetts, USA
Sucrose	Sigma-Aldrich	S0389	Burlington, Massachusetts, USA
Ethylenediaminetetraacetic acid (EDTA)	Sigma-Aldrich	E9884	Burlington, Massachusetts, USA
Mannitol	Sigma-Aldrich	M1902	Burlington, Massachusetts, USA
IGEPAL CA-630 (Nonidet™ P 40)	Supelco	56741	Burlington, Massachusetts, USA
Hoechst 33342	Thermo Fisher Scientific	62249	Waltham, Massachusetts, USA
DAPI	Thermo Fisher Scientific	62248	Waltham, Massachusetts, USA

Reagent Kit

Name	Brand	Catalog number	
Trans-Blot Turbo RTA Transfer Kit, PVDF	Bio-rad	1704272	Feldkirchen, Germany
Lentivirus Titer Kit, HIV-1 p24 ELISA	OriGene	TR30038	Rockville, Maryland, USA

Griess Reagent System	Promega	G2930	Madison, Wisconsin, USA
Clarity Max Western ECL Substrate	Bio-rad	1705062	Feldkirchen, Germany
SuperSignal™ West Atto Ultimate Sensitivity Substrate	Thermo Fisher Scientific	A38554	Waltham, Massachusetts, USA
RIPA Lysis Buffer System	Chem Cruz	sc-24948A	Dallas, Texas, USA
Hemin Assay Kit	Sigma-Aldrich	MAK316	Burlington, Massachusetts, USA
BC Assay Protein Quantitation Kit	Interchim	UP40840A	Montluçon, France
ELISA MAX™ Deluxe Set Mouse TNF-α	BioLegend	430904	San Diego, California, USA
ELISA MAX™ Deluxe Set Mouse IL-6	BioLegend	431304	San Diego, California, USA

Consumables

Name		Catalog number	
Millex®-GV Filter Unit (Sterile), 0.22 µm, PVDF	Merck	SLGV033RB	Darmstadt, Germany
Millex®-HP Filter Unit (Sterile), 0.45 µm, PES	Merck	SLHP033RB	Darmstadt, Germany
TC-plate, Cell culture, 96 well, Suspension, F	SARSTEDT	83.3924.500	Nümbrecht, Germany
TC-plate, Cell culture, 6 well, Suspension, F	SARSTEDT	83.3920.500	Nümbrecht, Germany
TC-plate, Cell culture, 12 well, Suspension, F	SARSTEDT	83.3921.500	Nümbrecht, Germany
TC-plate, Cell culture, 96 well, Standard, F	SARSTEDT	83.3924.005	Nümbrecht, Germany
Tissue Culture Test Plate, 12 well, Growth-enhanced treated	TPP	92012	Trasadingen, Schaffhausen, Switzerland
Tissue Culture Dish, 100 mm	TPP	93100	Trasadingen, Schaffhausen, Switzerland
Tube, 15 ML	greiner BIO-ONE	188271	Frickenhausen, Baden-Württemberg, Germany
Tube, 50 ML	greiner BIO-ONE	227261 - N	Frickenhausen, Baden-Württemberg, Germany
ELISA-Plate, High Binding, F	SARSTEDT	82.1581.200	Nümbrecht, Germany
96-Well Black Plate, F	Costar	3915	Corning, NY, USA
70 mL Polycarbonate Centrifuge Bottle	Beckman Coulter	355655	Brea, California, USA
Falcon 70 µm Cell Strainer	Corning	352350	Corning, NY, USA
BD Microlance™ 3, G22, 1 1/4"	BD Becton Dickinson	300900	Franklin Lakes, New Jersey, United States
MicroAmp™ Fast Optical 96-Well Reaction Plate, 0.1 mL	Applied Biosystems	4346907	Waltham, Massachusetts, USA

Software and Analytical Tools

Software	Company	Version	
GraphPad Prism	GraphPad Software	9.1.0	San Diego, CA, USA
ImageJ	National Institutes of Health	1.54f	Washington, USA
FlowJo	FlowJo LLC	10.8.1	Ashland, OR, USA
Image Lab	Bio-Rad	6.0.0	Hercules, California, USA
Gen5	Biotek	3.09.07	Winooski, Vermont, USA
NanoDrop 1000	Thermo Fisher Scientific	3.8.1	Waltham, Massachusetts, USA
StepOne Software	Thermo Fisher Scientific	2.3	Waltham, Massachusetts, USA
BD FACSDiva Software	BD Biosciences	8.0.2	San Jose, CA, USA
BZ-X800 Viewer	KEYENCE	1.3.0.1	Osaka, Japan
Magellan	TECAN	7.5	Männedorf, Switzerland
BioRender	BioRender		Toronto, ON, Canada
