# Supplementary material for: Dynamic alterations in labile heme levels and heme biosynthesis during inflammatory activation of macrophages
Source: Redox Biol. 2026 Jul 16;96:104305. doi: 10.1016/j.redox.2026.104305 (PMC13393713; doi:10.1016/j.redox.2026.104305)
Supplement: Multimedia component 2 [file mmc2.pdf]

SUPPLEMENTARY FIGURES

Figure S1

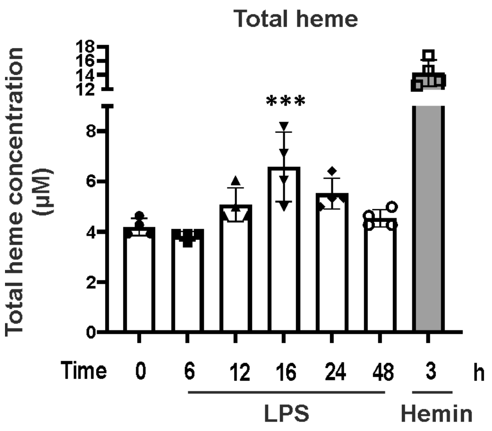

Figure S1. LPS increases total heme levels in BMDMs. Total heme levels were quantified using a heme assay kit in BMDMs treated with LPS (1 μg/ml) for indicated times, or with hemin (10 μM, 3h) as a positive control. Data represent mean ± SD ($n \geq 3$). Statistical analysis was performed using one-way ANOVA followed by Tukey's post-hoc analysis; ***$p < 0.001$ vs. Con.

Figure S2

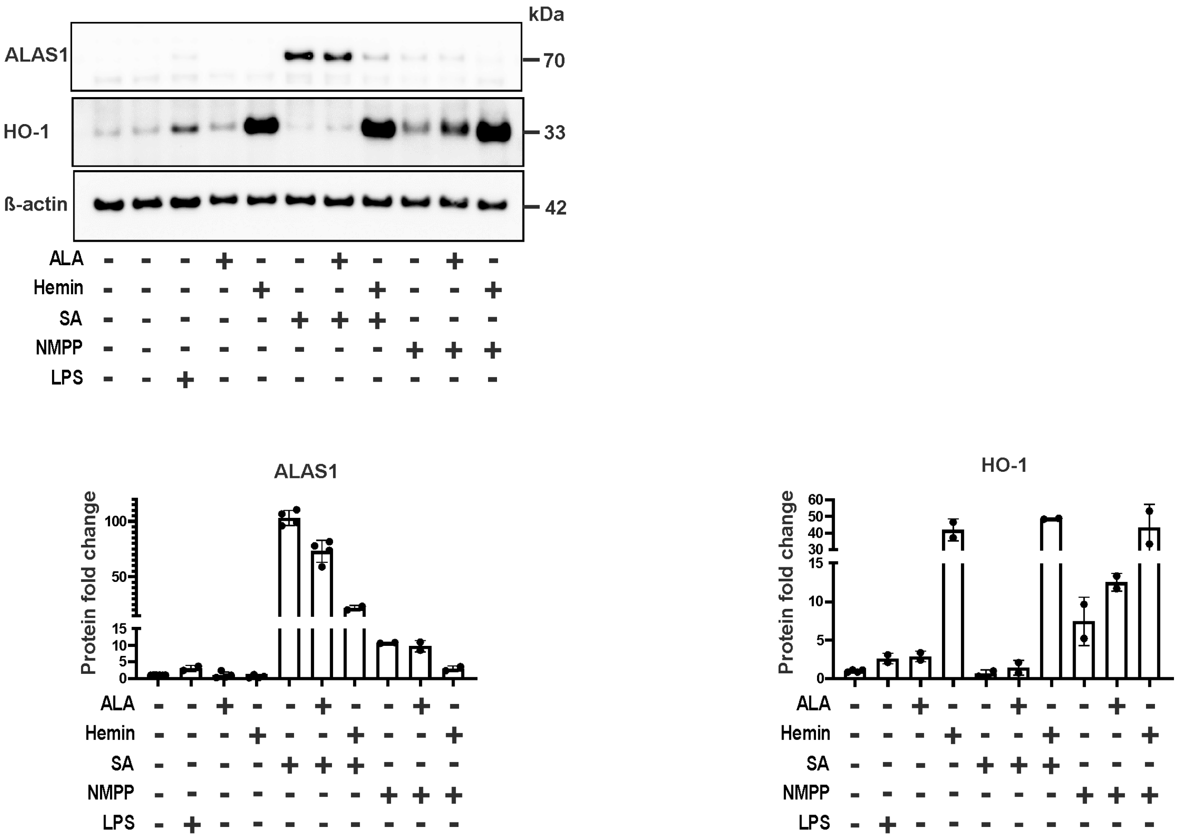

Figure S2. Intracellular LH levels regulate ALAS1 and HO-1 expression in inflammatory macrophages. (Top) Western blot analysis of ALAS1, HO-1 and β-actin in total lysates from BMDMs treated with SA (2mM), ALA (300μM) or NMPP (5μM) for 24 h, followed by LPS (1 μg/ml) or hemin (10 μM) for 16 h. (Bottom) Representative densitometric quantification normalized to β-actin. Data represent mean ± SD (n ≥ 2). ALA, δ-aminolevulinate; NMPP, N-methyl protoporphyrin; SA, succinylacetone.

Figure S3

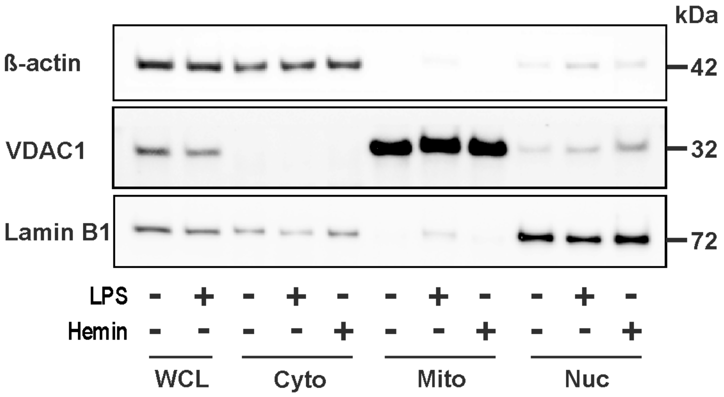

Figure S3. Verification of organelle isolation in BMDMs. Western blot analysis of VDAC1, lamin B1, and β-actin in whole-cell lysates, cytosolic, mitochondrial, and nuclear fractions from BMDMs treated with LPS (1 μg/ml) or hemin (10 μM) for 16 h. cyto, cytosol; mito, mitochondria; nuc; nucleus; WCL, whole-cell lysate.

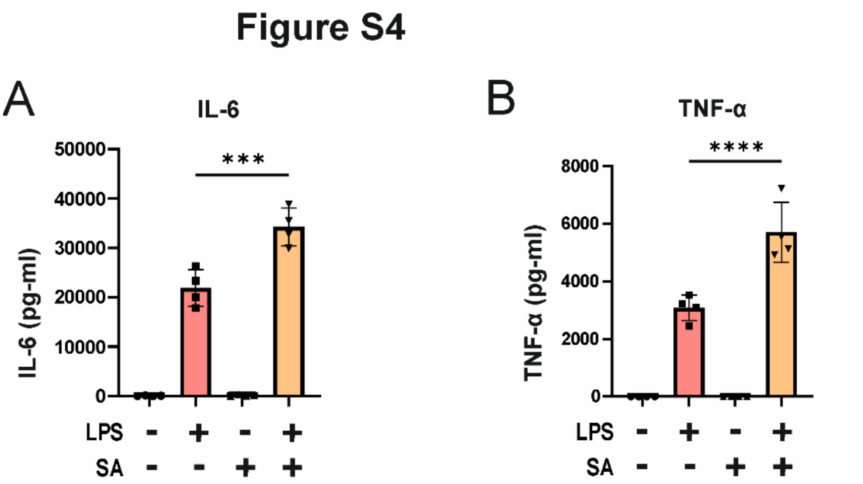

Figure S4. SA increases pro-inflammatory cytokine secretion in LPS-treated BMDMs. IL-6 (A) and TNF-α (B) concentrations in cell culture supernatants measured by ELISA. BMDMs were pretreated with SA (2 mM) for 24 h followed by LPS stimulation (1 μg/ml, 16 h). Data represent mean ± SD ($n \geq 3$). Statistical analysis was performed using one-way ANOVA followed by Tukey's post-hoc analysis; ***$p < 0.001$, ****$p < 0.0001$. SA, succinylacetone.

Figure S5

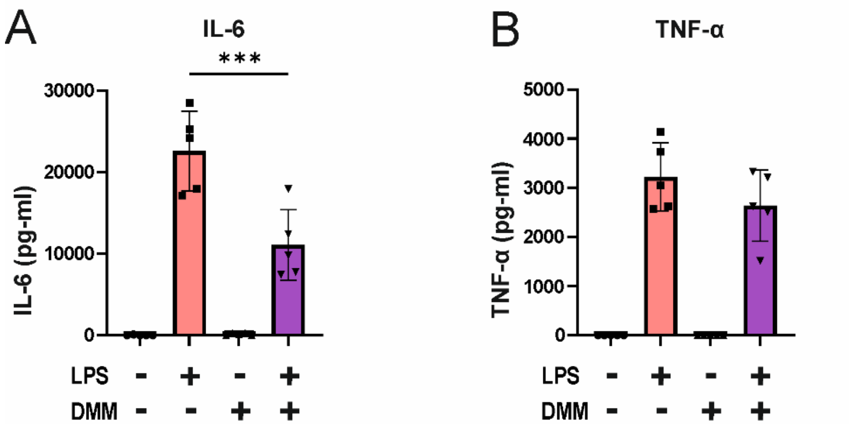

Figure S5. DMM modulates pro-inflammatory cytokine secretion in LPS-treated BMDMs. IL-6 (A) and TNF-α (B) concentrations in cell culture supernatants measured by ELISA. BMDMs were pretreated with DMM (5 mM) for 24 hours followed by LPS stimulation (1 µg/ml, 16 h). Data represent mean ± SD ($n \geq 3$). Statistical analysis was performed using one-way ANOVA followed by Tukey's post-hoc analysis; ***$p < 0.001$. DMM, dimethyl malonate.
